# PDE5 Inhibitors-Loaded Nanovesicles: Physico-Chemical Properties and *In Vitro* Antiproliferative Activity

**DOI:** 10.3390/nano6050092

**Published:** 2016-05-18

**Authors:** Roberta F. De Rose, Maria Chiara Cristiano, Marilena Celano, Valentina Maggisano, Ada Vero, Giovanni Enrico Lombardo, Martina Di Francesco, Donatella Paolino, Diego Russo, Donato Cosco

**Affiliations:** 1 Department of Health Sciences, University “Magna Græcia” of Catanzaro, Campus Universitario “S. Venuta”, Viale S. Venuta, Germaneto, Catanzaro I-88100, Italy; robertafra87@gmail.com (R.F.D.R.); mchiara.cristiano@unicz.it (M.C.C.); celano.donato@tiscali.it (M.C.); vale.fede1@virgilio.it (V.M.); adavero@hotmail.it (A.V.); gelombardo@unicz.it (G.E.L.); martina.difrancesco89@gmail.com (M.D.F.); 2 Department of Experimental and Clinical Medicine, University “Magna Græcia” of Catanzaro, Campus Universitario “S. Venuta”, Viale S. Venuta, Germaneto, Catanzaro I-88100, Italy; paolino@unicz.it

**Keywords:** phosphodiesterase-5 (PDE5) inhibitors, nanoliposomes, human thyroid carcinoma, drug delivery systems

## Abstract

Novel therapeutic approaches are required for the less differentiated thyroid cancers which are non-responsive to the current treatment. In this study we tested an innovative formulation of nanoliposomes containing sildenafil citrate or tadalafil, phosphodiesterase-5 inhibitors, on two human thyroid cancer cell lines (TPC-1 and BCPAP). Nanoliposomes were prepared by the thin layer evaporation and extrusion methods, solubilizing the hydrophilic compound sildenafil citrate in the aqueous phase during the hydration step and dissolving the lipophilic tadalafil in the organic phase. Nanoliposomes, made up of 1,2-dipalmitoyl-sn-glycero-3-phosphatidylcholine monohydrate (DPPC), cholesterol, and *N*-(carbonyl-methoxypolyethylene glycol-2000)-1,2-distearoyl-sn-glycero-3-phosphoethanolamine (DSPE-mPEG2000) (6:3:1 molar ratio), were characterized by a mean diameter of ~100 nm, a very low polydispersity index (~0.1) and a negative surface charge. The drugs did not influence the physico-chemical properties of the systems and were efficiently retained in the colloidal structure. By using cell count and MTT assay, we found a significant reduction of the viability in both cell lines following 24 h treatment with both nanoliposomal-encapsulated drugs, notably greater than the effect of the free drugs. Our findings demonstrate that nanoliposomes increase the antiproliferative activity of phosphodiesterase-5 inhibitors, providing a useful novel formulation for the treatment of thyroid carcinoma.

## 1. Introduction

The prevalence of thyroid carcinomas is increasing worldwide [1,2]. While the majority of these tumors are well differentiated and may be efficaciously managed by using the radioiodine treatment after surgery, there is a minority of less-differentiated carcinomas which do not respond to the standard treatment [3,4,5]. This behavior is aggressive and is owed to the oncogenic activation of intracellular signal transduction pathways responsible for the dysregulation of both cell proliferation and differentiation [6,7,8,9]. The discovery of the molecular alterations influencing the intracellular messengers in thyroid tumor cells has allowed us to identify novel molecular targets and to select a series of novel agents able to act against these molecular targets. We have recently demonstrated the overexpression of phosphodiesterase-5 (PDE5) that occurs, the enzyme which specifically regulates the intracellular levels of cGMP, in a series of thyroid carcinomas; also, the inhibition of PDE5 by sildenafil (SIL) or tadalafil (TAD) determined a block in the proliferation of thyroid cancer cells in culture, suggesting that specific inhibitors of PDE5 may be proposed for the treatment of these tumors [10]. Recent studies have demonstrated that PDE5 is involved in the progression of different cancers (breast, colorectal, prostate) which makes it an important molecular target for anti-cancer therapy [11,12,13].

It is a well-established fact that the encapsulation of drugs in biocompatible nanocarriers allows the modulation of their biopharmaceutical properties, thus, favouring the increase of their pharmacological activity and the decrease of the side effects [14,15]. Liposomes are drug delivery systems able to entrap lipophilic, hydrophilic, and amphilphilic compounds and to target specific areas as a consequence of their physico-chemical and technological characteristics [16,17]. The encapsulation of doxorubicin in 100 nm-liposomes (*i.e.*, Doxil, Caelyx), for example, generated a colloidal formulation used in many clinical protocols as a treatment of election for different tumors [18,19]. In fact, the anatomy of the blood vessels of the tumor, characterized by broad fenestrations, together with the peculiar osmotic pressure of the microenvironment give rise to the so-called Enhanced Permeability and Retention (EPR) effect that allows the localization of the colloids in the solid tumor mass [20,21]. To the best of our knowledge, no liposomal formulation containing a PDE5 inhibitor has been developed for systemic administration by the iv route [22,23,24,25,26]. Herein, PEGylated nanoliposomes containing SIL or TAD (SIL-nlip or TAD-nlip, respectively) have been developed, characterized, and investigated in order to evaluate their antiproliferative effect on human thyroid carcinoma cells with respect to the free drug(s).

## 2. Results

### 2.1. Physico-Chemical Characterization of Nanoliposomes Containing PDE5 Inhibitors

**Figure 1:**
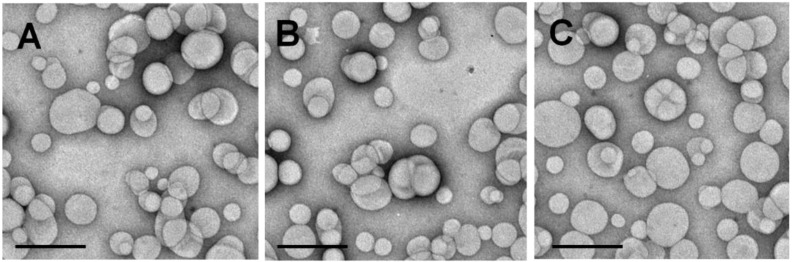
Transmission electron microscopy (TEM) micrographs of empty nanoliposomes (**A**); SIL-nanoliposomes (**B**) and TAD-nanoliposomes (**C**). Bar: 200 nm.

Specific parameters including shape, size, surface charge and lamellarity strongly influence the biological characteristics of liposomes. For this reason, we first analyzed the physico-chemical properties of the vesicles [27,28]. The DLS analysis evidenced a mean diameter of the empty vesicles of about 100 nm, a homogeneous size distribution and a surface charge of ~−20 mV, thus confirming the data previously reported by our research team [16] (Table 1). The encapsulation of SIL did not induce a dramatic change in the mean sizes while the lipophilic TAD favored a slight increase in the colloidal diameter and of the polydispersity index (PDI) value. This phenomenon could be closely related to the liposomal localization of the drug which could induce a certain destabilization of the vesicular bilayer. Transmission Electron Microscopy (TEM) analysis confirmed the DLS data (Figure 1). TAD did not modify the surface charge of the nanosystems while SIL induced a decrease in the Zeta potential, probably as a consequence of its interaction/adsorption with the PEG moieties of the nanovesicles (Table 1 and Table 2). This trend was not surprising, since our research team previously demonstrated that this occurs when the salt derivative of an active compound is entrapped within nanoliposomes made up of the same lipid mixture used in this investigation [29,30].

We next investigated the drug-loading capacity in order to evaluate the amount of PDE5 inhibitors retained by the vesicular structure: the encapsulation efficiency of the two drugs were quite different as a consequence of their physico-chemical characteristics. In detail, TAD was well retained in the lipophilic compartment of the nanoliposomes providing a value of ~81%, while SIL was retained in the aqueous compartment at ~49%.

**Figure 2:**
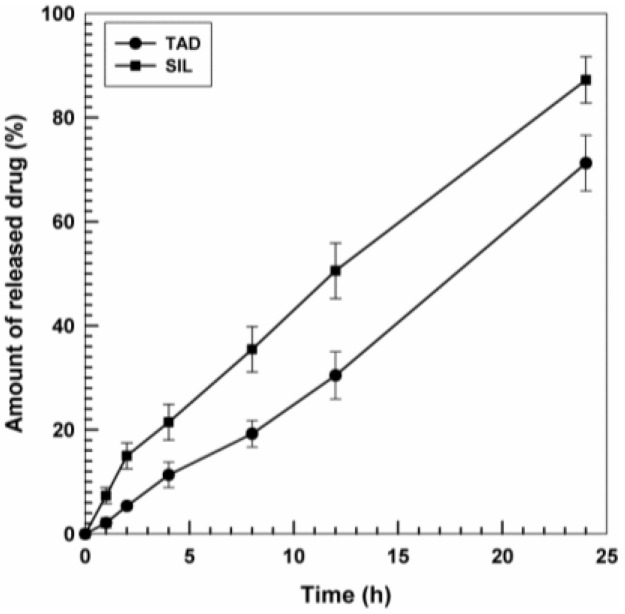
Release profile of SIL and TAD from nanoliposomes. Experiments were carried out at room temperature. Values were the mean of three independent experiments in triplicate ± standard deviation (SD).

Drug release was closely related to the physico-chemical properties of the compounds; in fact, nanoliposomes showed a slow and prolonged release profile of TAD characterized by a leakage of the active compound of less than 20% after the first 8 h and a continuous release up to 24 h, which allowed a TAD efflux of ~65% (Figure 2). On the contrary, the release of SIL was characterized by a rapid drug leakage after 4 h (~20%) and a prolonged one up to 80% after 24 h (Figure 2).

### 2.2. Effects of Nanoliposomes Containing PDE5 Inhibitors on Thyroid Cancer Cells

For the initial screening of the effects of these novel formulations, we employed a widely-used *in vitro* experimental model, represented by two cell lines derived from human papillary thyroid carcinoma, TPC-1, and BCPAP cells, carrying a RET/PTC or BRAFV600E genotypic alteration, respectively [31].

**Figure 3:**
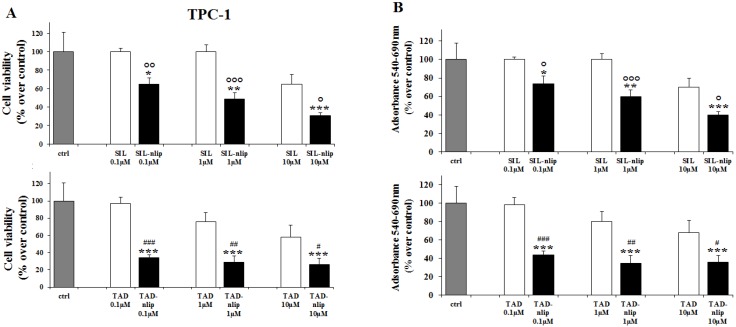
Nanoliposomes containing PDE5 inhibitors reduced proliferation of TPC-1 cells. After 24 h of treatment, proliferation was evaluated by cell count assay (**A**) and MTT assay (**B**), as described in Materials and Methods. Results are mean ± SD of three experiments performed in triplicate. *, **, ***, *p* < 0.05, 0.01, 0.001 *vs.* control (ctrl); °, °°, °°°, SIL-nlip *vs* SIL; ^#^, ^##^, ^###^, *p* < 0.05, 0.01, 0.001 TAD-nlip *vs.* TAD.

**Figure 4:**
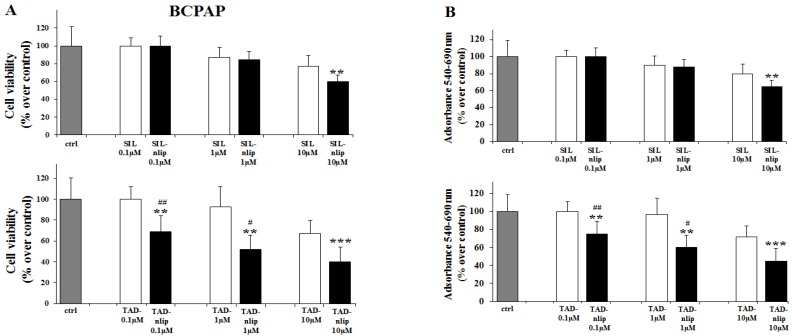
Nanoliposomes containing PDE5 inhibitors reduced proliferation of BCPAP cells. After 24 h of treatment, proliferation was evaluated by cell count assay (**A**) and MTT assay (**B**), as described in Materials and Methods. Results are mean ± SD of three experiments performed in triplicate. *, **, ***, *p* < 0.05, 0.01, 0.001 *vs.* control (ctrl); ^#^, ^##^, *p* < 0.05, 0.01 TAD-nlip *vs.* TAD.

The effects of SIL and TAD on the proliferation of TPC-1 and BCPAP were first evaluated by cell counting, comparing the free drugs with the molecules encapsulated in the nanovesicular carrier. As shown in Figure 3 and Figure 4, we observed a slight decrease in TPC-1 and BCPAP cells treated for 24 h with SIL and TAD at a concentration of 10 µM. When the drugs were encapsulated, a significant effect on the proliferation was observed even at 0.1 µM in TPC-1 cells compared to both the control (SIL-nlip *p* < 0.05; TAD-nlip *p* < 0.001) and the free drug (SIL-nlip *p* < 0.01; TAD-nlip *p* < 0.01), while in BCPAP a much more significant reduction of cell growth appeared only with TAD encapsulated in nanoliposomes at a concentration of 0.1 µM (*p* < 0.01) (Figure 4A). Similar results were observed with the MTT assay (Figure 3B and Figure 4B). It can be hypothesized that a specific genetic background (in particular, BRAF V600E mutation in BCPAP) had made the cells more or less resistant to the action of the free drugs, even to the point of influencing their response to the different inhibitors. In this regard, it was thanks to drug encapsulation that a significant antiproliferative effect could be observed in both cell lines treated with both inhibitors.

**Figure 5:**
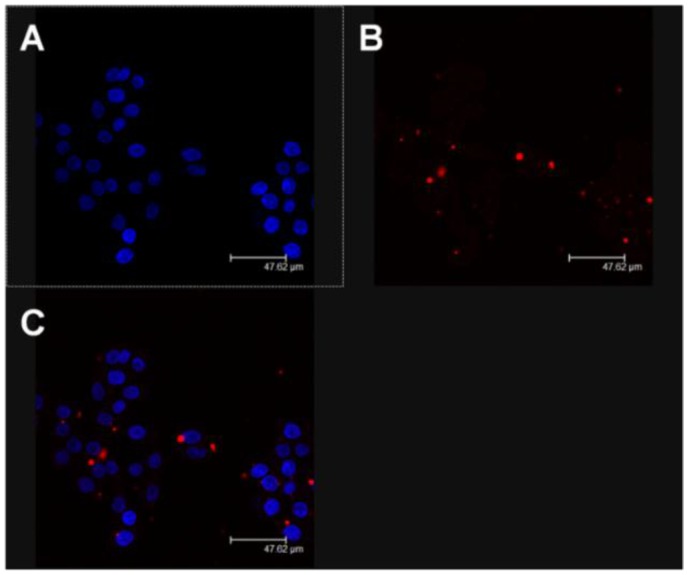
Confocal laser scanning microscopy (CLSM) micrographs of TPC-1 cells treated with rhodamine-labelled nanoliposomes after 3 h incubation: **panel A**, Hoechst filter; **panel B**, TRITC filter; and **panel C**, overlay. A representative example of three independent experiments.

**Figure 6:**
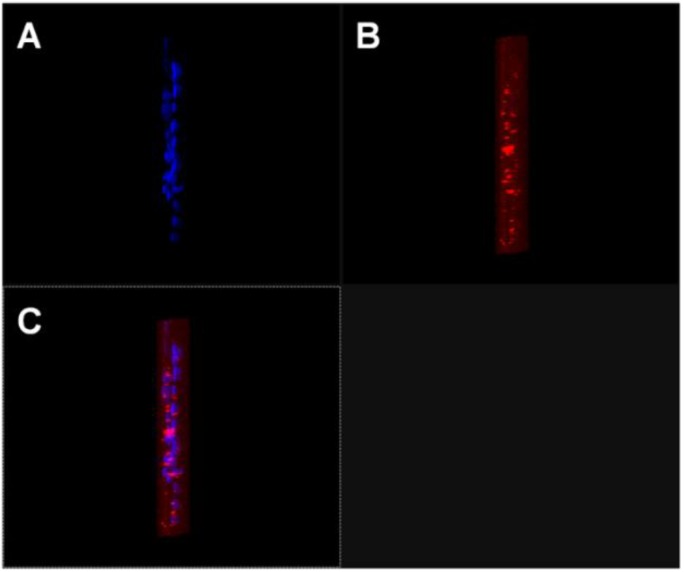
Z-stack analysis by CLSM of TPC-1 cells treated with rhodamine-labelled nanoliposomes after 3 h incubation: **panel A**, Hoechst filter; **panel B**, TRITC filter; and **panel C**, overlay. A representative example of three independent experiments.

A reasonable explanation for the aforesaid trend could be related to the amply described properties of vesicular colloidal carriers that are able to increase the localization of the entrapped active compounds inside cells. For this reason, a confocal laser scanning microscopy (CLSM) analysis was performed in order to investigate the interaction rate between the fluorescent nanosystems and TPC-1 cells. In Figure 5 the significant interaction of the colloidal systems with cells after 3 h incubation can be observed; in detail, the red spots, deriving from the integration of the rhodamine-phospholipids in the liposomal structure, demonstrates the onset of the cellular uptake of the nanoliposomes. It must be recognized, however, that a 2D visualization cannot certify the real intracellular localization of the nanosystems because other kinds of interaction phenomena could be involved (such as adsorption, lipid exchange, *etc.*). For this reason, a Z-stack analysis was performed furnishing clear evidence of the massive intracellular localization of the fluorescent nanosystems (Figure 6). This experiment corroborates the hypothesis that the increased pharmacological activity of the active compounds is closely related to their efficient localization in the cells promoted by their nanoencapsulation, which allows the reduction of the efficacious dosage with respect to the free form.

## 3. Discussion

The improvement of the pharmacological effect of antitumor compounds is an important goal in modern anticancer therapy. The selective targeting of specific body compartments can be useful for increasing the efficacy of antitumor drugs and decreasing undesirable side effects in patients. In this context, the use of colloidal systems able to deliver active compounds is a valuable strategy that allows (i) the administration of molecules with different physico-chemical properties; (ii) the modulation of their biopharmaceutical properties and (iii) the increase of their specific tissue localization as a consequence of the technological characteristics of the carrier [16]. To the best of our knowledge, the PDE5 inhibitors TAD and SIL have never been encapsulated within a vesicular colloidal system for systemic administration with the aim of treating thyroid cancer-related diseases. To date, in fact, they have only been entrapped in lipid-based systems in order to obtain efficient transdermal delivery [22,23] or in polymer-based micro- and nanosystems with the aim of treating pulmonary hypertension [24,25,26]. The specific diameter of the liposomes herein described, and the coating of the vesicles with PEG, are fundamental factors for developing a nanoformulation characterized by long circulation properties [32]. Even though the multiple administration of PEG-coated nanosystems gives rise to the appearance of the so-called accelerated blood clearance (ABC) phenomenon, this polymer remains the best compound for increasing the plasmatic half-life of nanosystems [33].

The present findings demonstrate that nanoformulations containing SIL and TAD may be potential innovative nanomedicines for use in the treatment of those thyroid carcinomas unresponsive to current treatments. In fact, we demonstrated that the encapsulation of these compounds within liposomes allowed a significant decrease of the drug dosages necessary to effectively reduce thyroid carcinoma cell proliferation. Moreover the efficacy of the encapsulated drug even in those cancer cells carrying a genetic alteration associated with an aggressive behavior [34,35] may have an important implication for potential personalized therapeutic approaches. At present, there are some reports which suggest the potential use of PDE5 inhibitors as antineoplastic agents [10,36,37], but the possible impact of adverse dose-dependent effects owing to their vasodilatatory action has not yet been investigated. If confirmed even *in vivo*, such a property of our new formulations which would permit the adoption of a low-dosage regimen of the drugs could help to overcome this fundamental issue. The aforesaid trend is well known in clinical practice because liposome-based formulations containing anticancer drugs are widely used in many protocols [38]. Moreover, nanoliposomes may allow the co-encapsulation of the two compounds or their association with other antitumor drugs [39,40] in the same nanodevice and favor a further improvement of their pharmacological efficacy as a consequence of a suitable multidrug delivery. Future studies will furnish further details on the effects of our novel drug devices and better clarify the validity of this approach for the treatment of thyroid tumors.

## 4. Experimental Sections

### 4.1. Chemicals

The phospholipids used for the preparation of liposomes, 1,2-dipalmitoyl-sn-glycero-3-phosphatidylcholine monohydrate (DPPC) and the *N*-(carbonyl-methoxypolyethylene glycol-2000)-1,2-distearoyl-sn-glycero-3-phosphoethanolamine (DSPE-mPEG2000) were provided by Avanti Polar Lipids (Spectra 2000, Rome, Italy). The cholesterol (Chol), sildenafil citrate (SIL) and tadalafil (TAD) were purchased from Sigma Aldrich (Milan, Italy). D-MEM culture medium, fetal bovine serum (FBS), trypsin-EDTA (1×) solution, penicillin-streptomycin solution, and Lissamine rhodamine B 1,2 dihexadecanoyl-sn-glycero-3-phosphoethanolamine triethylammonium salt (rhodamine DHPE) were obtained from Invitrogen (Life Technologies, Monza, Italy). Human thyroid cancer cell lines (TPC-1 and BCPAP) were provided by Prof. G. Damante (University of Udine) and Prof. E. Puxeddu (University of Perugia). All other materials and solvents used in this investigation were of analytical grade (Carlo Erba, Milan, Italy).

### 4.2. Preparation of Nanoliposomes Containing PDE5 Inhibitors

The liposomes were prepared by the thin-layer-evaporation method as previously described [39]. Briefly, a lipid mixture (20 mg) made up of DPPC:Chol:DSPE-mPEG2000 (6:3:1 molar ratio) was dissolved in 1 mL of chloroform/methanol (3:1 *v*/*v*). Successively, the solvent was removed by means of a rotavapor Büchi R-210 (Flawil, Switzerland) in order to obtain the formation of a film layer on the inner walls of the tube, and overnight storage at room temperature in a Büchi T51 glass drying oven connected to a vacuum pump. The samples were hydrated with 1 ml of bi-distilled water or NaCl (0.9% *w*/*v*) aqueous solution and submitted to three alternate cycles (3 min each) of warming to 58 °C in a thermostated water bath and vigorous mixing by vortex at 11× *g*. The resulting multilamellar liposomes were kept at 57–60 °C for 3 h to anneal the bilayer structure. Considering the physico-chemical characteristics of PDE5 inhibitors, TAD was added in the organic phase (1 mg), while SIL (1.5 mg) was added to the aqueous phase during the preparation of the nanoliposomes (Table 2). Successively, multilamellar vesicles were extruded by a Lipex Extruder (Vancouver, BC, Canada) through polycarbonate membrane filters (Nucleopore^®^ Polycarbonate) in order to decrease their mean diameter and to obtain devices suitable for systemic administration [32].

Fluorescent liposomes were prepared by co-dissolving rhodamine-DHPE (0.1%) with the lipid mixture.

### 4.3. Physico-Chemical and Technological Characterization of Nanoliposomes

Mean size, size distribution and z-potential of nanosystems were evaluated by a dynamic light scattering spectrophotometer Zetasizer Nano ZS (Malvern Instruments Ltd., Worchestershire, UK), while their morphology was investigated by means of a transmission electron microscope (Philips, Eindhoven, The Netherlands) as previously described [41].

### 4.4. Drug Entrapment Efficiency and Release Profile

The amount of TAD and SIL entrapped in the vesicular structure was investigated using a suitable spectrophotometric method. In particular, the liposomal suspension containing the PDE5 inhibitors was ultra-centrifuged (80k× *g* for 1 h at 4 °C) using a Beckman Optima™ ultracentrifuge (Rome, Italy) with a TL S55 fixed-angle rotor. The pellet was mixed with methanol in order to disrupt the vesicular structure and analyzed by a spectrophotometer (Perkin Elmer Lambda 35, Waltham, MA, USA) at the λ_max_ of 280 nm and 290 nm for TAD and SIL, respectively. SIL required the addition of water in order to allow the solubilization of the active compound. No interference peaks deriving from the liposomal components were observed during the spectrophotometric investigation (an empty liposomal formulation was used as a blank).

The amount of drug contained within the nanoliposomes was determined as the difference between the amount of active compounds added during sample preparation and the amount of the drug detected afterwards through spectrophotometric analysis. The entrapment efficiency (*EE* %) was calculated with the following equation:

$$EE\;\%=\frac{De}{Da}\times 100$$

where *De* is the amount expressed in mg of entrapped drug and *Da* is the amount expressed in mg of drug added during the preparation of the nanosystems.

The drug release profiles were investigated by the dialysis method using cellulose acetate dialysis tubing (Spectra/Por with molecular cut-off 12,000–14,000 by Spectrum Laboratories Inc. Breda, The Netherlands) sealed at both ends with clips as previously described [40]. A pH 7.4 phosphate buffer solution/ethanol (70:30 *v*/*v*) was used as receptor fluid. A sample of release fluid (1 mL) was withdrawn and replaced with the same volume of fresh fluid after different incubation times. Samples were then spectrophotometrically analyzed at the λ_max_ previously mentioned.

The percentage of released drug was calculated using the following equation:

Release (%) = drug_rel_/drug_load_ × 100

where drug_rel_ is the amount of drug released at the time *t* and drug_load_ is the amount of drug entrapped within nanoliposomes. The release studies were carried out in triplicate.

### 4.5. Thyroid Cancer Cell Lines

Human thyroid carcinoma cells TPC-1 and BCPAP, carrying well-defined genotypic alterations [28], were cultured in DMEM or RPM-1 medium, respectively, as previously described [10].

### 4.6. Cell Viability Assay

For the cell viability assay, TPC-1 and BCPAP were seeded in 12-well plates at a density of 20 × 10^3^ or 50 × 10^3^ cells/well. After 24 h, fresh normal medium was supplemented with SIL, TAD and liposomes containing the single drug at different doses, (0.1, 1, 10 µM) or equivalent dilutions of dimethyl sulfoxide (control). Cells were incubated for 24 h and then were trypsinized and counted in counter slides (Countess, Thermo Fisher Scientific, Milano, Italy). Proliferation was also evaluated with the MTT assay [42]. Cells were seeded at a density of 3.5 × 10^3^ (TPC-1) and 5 × 10^3^ (BCPAP) into 96-well plates in 100 µL of medium. Twenty-four hours later, the cells were treated and then incubated for 24 h. The solubilized formazan product was quantified using a microplate spectrophotometer (X-MARK, Bio-Rad, Milano, Italy) at a wavelength of 540 nm and a reference wavelength of 690 nm.

### 4.7. Confocal Laser Scanning Microscopy (CLSM) Analysis

The interaction between the cells and nanoliposomes was evaluated through CLSM studies as previously described [40]. Briefly, the cells (4 × 10^5^ cells/mL) were placed in six-well culture plates with culture medium and a sterile glass slide was positioned in each well. The plates were incubated for 24 h and then the cells were treated with rhodamine-labeled vesicular nanocarriers for 6 h. After incubation, each well was washed 3 times with PBS to remove the excess nanoliposomes and the cells were fixed on the sterile glass slides using 1 mL of a 70% *v*/*v* ethanol solution. Each slide was again washed three times with PBS and 2 mL of PBS was added to each well. The plates were stored at 4 °C up to the moment of CLSM analysis. Before analysis, cover-slides were positioned over the glass slides using a 70% *v*/*v* glycerol solution to remove enclosed air and then fixed with a transparent glue. The analysis was carried out using a Leika TCS SP2 MP laser scanning confocal microscopy operating at λ_exc_ = 560 nm and λ_em_ = 580 nm for the rhodamine probe and at λ_exc_ = 405 nm and λ_em_ = 460 nm for the Hoechst probe. A scan resolution of up to 1024 × 1024 pixels with an Ar/Kr laser beam of 75 mW equipped with a TRITC analyzer filter was used for experimental investigation. Sample micrographs were recorded by a macro developer software package having multi-dimensional series acquisition and direct-access digital control knobs. An immersion oil lens with 63× magnification was used.

### 4.8. Statistical Analysis

Results are expressed as mean ± standard deviation (SD). For cell proliferation assays the one-way ANOVA followed by the Tukey-Kramer multiple comparisons test was adopted. *p*-values lower than 0.05 were considered statistically significant. All statistical analyses were performed using GraphPad Prism version 5.0 statistical software (GraphPad Software Inc., San Diego, CA, USA).

## Figures and Tables

**Table 1:** Physico-chemical parameters of nanoliposomes. ^a^

Sample	Mean Sizes (nm)	Polydispersity Index	Zeta Potential (mV)
Empty nlip ^b^	104.0 ± 1.5	0.05 ± 0.01	−18.5 ± 1.5
SIL-nlip ^c^	91.9 ± 2.7	0.09 ± 0.08	−7.3 ± 1.2
TAD-nlip ^d^	123.1 ± 0.9	0.121 ± 0.02	−19.7 ± 1.7

^a^ Each value represents the mean ± standard deviation of at least three experiments; ^b^ Empty nlip: Empty nanoliposomes; ^c^ SIL-nlip: sildenafil citrate nanoliposomes; ^d^ TAD-nlip: tadalafil nanoliposomes.

**Table 2:** Physicochemical properties of sildenafil citrate and tadalafil used for encapsulation within nanoliposomes.

Parameters	Sildenafil Citrate	Tadalafil
2D structure	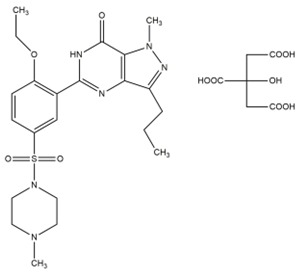	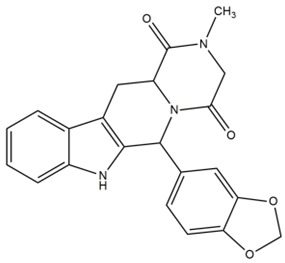
logP	1.9 (free base)	1.7
Melting point (°C)	189–190 (free base)	301–302
Molecular formula	C_28_H_38_N_6_O_11_S	C_22_H_19_N_3_O_4_
Molecular weight	666.69	389.4
